# Effects of vitamins E and C combined with β-carotene on cognitive function in the elderly

**DOI:** 10.3892/etm.2015.2274

**Published:** 2015-02-09

**Authors:** YONGHUA LI, SHUMEI LIU, YIGANG MAN, NING LI, YU ZHOU

**Affiliations:** 1 Department of Nutrition and Food Hygiene, Jining Medical College, Jining, Shandong 272013, P.R. China; 2 Department of Dermatology, Jining No. 1 People’s Hospital, Jining, Shandong 272011, P.R. China; 3 Department of Pediatrics, Jining No. 1 People’s Hospital, Jining, Shandong 272011, P.R. China

**Keywords:** vitamin E, vitamin C, β-carotene, elderly cognitive function, amyloid-β, estradiol

## Abstract

The aim of the present study was to investigate the effect of vitamins E (VE) and C (VC), combined with β-carotene (β-C), on cognitive function in the elderly. A total of 276 elderly subjects completed the prospective study following treatment with VE, VC and different doses of β-C or with VE only. Cognitive function was assessed by the Mini-Mental State Examination (MMSE) and Hasegawa Dementia Scale (HDS) tests. The plasma levels of amyloid-β (Aβ) and estradiol (E_2_) were determined by radioimmunoassay (RIA). Results from the MMSE and HDS assessments indicated that the treatment strategy of VE and VC combined with β-C significantly improved cognitive function in the elderly subjects, particularly with higher doses of β-C. Furthermore, RIA suggested that treatment with these vitamins could markedly reduce plasma Aβ levels and elevate plasma E_2_ levels. The present findings suggest that treatment with VE, VC and β-C results in promising improvements in cognitive function in the elderly.

## Introduction

Cognitive function is the intellectual process of understanding and reflecting on objective things in the human brain, and is affected by numerous factors, including gender, age, health status and genetic background (1). Cognitive impairment is one of the important early clinical features of several senile dementias, including Alzheimer’s disease (AD), a progressive degenerative disease of the central nervous system (2,3). The etiology of AD has yet to be fully understood, and there are several hypotheses regarding the disease pathogenesis, such as the free-radical, amyloid cascade, genetic and apolipoprotein E hypotheses, abnormal cell metabolism, apolipoprotein gene polymorphisms and environmental toxicity poisoning (4,5).

In recent years, the investigation of free radical toxicity and the antioxidant protection system in cognitive dysfunction has become a focus for research, particularly regarding the pathogenesis of AD (6). Studies on the effects of antioxidant vitamins on cognitive function in elderly individuals have predominantly been cross-sectional epidemiological surveys (7,8); however, there are currently few long-term nutritional intervention studies concerning the effects of combined antioxidant vitamins on cognitive function in the elderly and the association between the treatments and the plasma levels of disease markers (9,10).

In the present study, the effect of supplementation of antioxidant vitamins on cognitive function in the elderly was investigated. The elderly subjects were treated with vitamins E (VE) and C (VC), combined with different doses of β-carotene (β-C), for 16 weeks. The cognitive function of the subjects was assessed, and the plasma levels of amyloid-β (Aβ) and estradiol (E_2_) were determined. The association between the treatment and the disease markers, as well as the possible mechanisms, was discussed.

## Materials and methods

### Subjects

A total of 300 elderly subjects were included in this study, including 128 males and 172 females, aged 60–75 years. None of the subjects had a history of major diseases, and all were capable of self-caring, with normal consciousness. Any type of vitamin supplementation was not allowed within the two-month period prior to the study. Prior written and informed consent was obtained from every subject, and the study was approved by the Ethics Review Board of Jining Medical College (Jining, China). Data regarding the background information of the subjects included gender, age, educational level, health status and history of smoking and drinking.

The subjects were randomly divided into the five groups A-E (n=60 for each group). Groups A, B, C and D underwent daily administration of 200 mg VE (Qingdao Double Whale Pharmaceutical Co. Ltd., Qingdao, China) and 300 mg VC (Shandong Lukang Cisen Pharmaceutical Co. Ltd., Jining, China), combined with 16.7, 8.4, 5.6 or 0 mg/day β-C (Chengdu Tonglian Pharmaceutical, Co., Ltd., Chengdu, China), respectively, for 16 weeks consecutively. For group E, 5 mg/day VE was administered as a control. During the period of vitamin treatment, the diet and life styles of the subjects remained basically unchanged. The test conditions and dosing responses were observed and recorded carefully, and follow-ups were conducted for each patient every two weeks (telephone inquiries and visits).

### Cognitive function assessments

Mini-mental state examination (MMSE; 11) and Hasegawa Dementia Scale (HDS; 12) tests were used to assess the cognitive function of the elderly subjects. These assessments were performed by experienced and specially trained medical staff, with standardized questionnaires. The time and place of the investigation were in accordance with uniform standards, and each subject was assessed by the same investigator prior and subsequent to the treatment intervention.

### Blood sampling

Blood sample collection was performed prior and subsequent to the treatment intervention in the morning, when the subjects were in a fasted state. Venous blood (5 ml) was immediately collected into sterile heparin tubes placed on ice packs. The fresh anticoagulated blood was subjected to centrifugation at 1,500 × g for 5 min at <10°C, and the plasma was collected into 5-ml tubes and stored in a refrigerator at −80°C until testing.

### Radioimmunoassay (RIA)

Plasma levels of Aβ were detected using β-AP Ria kits from the Technology Development Center, Chinese People’s Liberation Army General Hospital (Beijing, China) and those of E_2_ using Estradiol ELISA kits from the Beijing Institute of Biotechnology (Beijing, China), according to the manufacturers’ instructions. The results were read using a γ counter (Cobra 5002/5003; Packard Instrument Co., Meriden, CT, USA).

### Statistical analysis

Data are expressed as the mean ± standard deviation. SPSS software, version 12.0 (SPSS, Inc., Chicago, IL, USA) was used for the statistical analysis. Analysis of variance, the Student’s t-test and the χ^2^ test were performed for the comparisons. P<0.05 was considered to indicate a statistically significant difference.

## Results

### Background information and baseline analysis of the elderly subjects

The background information and baseline conditions of the subjects were first determined and analyzed. Out of a total of 300 elderly subjects, 276 received and completed the treatments with nutritional supplements (116 male and 160 female, aged 67.06±5.33 years). The remaining 24 subjects did not complete the study due to various reasons: One subject succumbed, 13 subjects moved elsewhere and 10 subjects quit the test, resulting in a loss of follow-up rate of 8% (24/300). As presented in Table I, no significant differences were observed in the gender and age (according to analysis of variance) or in the educational level (analyzed by χ^2^ test) among the groups (P>0.05). The percentages of the subjects with a smoking and/or drinking history were relatively high among the groups (35–57%). Subjects with high blood pressure and/or chronic bronchitis accounted for ~10% in each group. According to the χ^2^ test, there were no significant differences in smoking and drinking history or health status among the groups (P>0.05; Table II). These results suggest that the elderly subjects were suitable for the assessment and investigation, and that the grouping was also appropriate.

### Effect of VE and VC combined with β-C on the cognitive function of the elderly subjects

**Figure 1:**
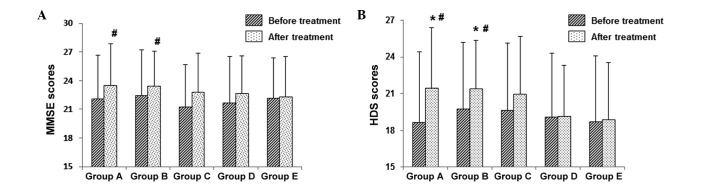
Cognitive assessments of the elderly subjects prior and subsequent to treatment. (A and B) Assessment of the cognitive function of the elderly subjects prior and subsequent to treatment with VE, VC and β-C using (A) the MMSE and (B) the HDS tests. Groups A–D underwent daily administration of 200 mg VE + 300 mg VC, combined with 16.7, 8.4, 5.6 or 0 mg/day β-C, respectively. For group E, 5 mg/day VE was administered as a control. Compared with the score prior to the treatment, ^*^P<0.05; compared with the score in group E, ^#^P<0.05. MMSE, Mini-Mental State Examination; HDS, Hasegawa Dementia Scale; VE, vitamin E; VC, vitamin C; β-C, β-carotene.

To investigate the effect of VE and VC combined with β-C on the cognitive function of the elderly subjects, the MMSE and HDS tests were used for the assessments. The subjects from groups A-D underwent daily administration of 200 mg VE + 300 mg VC, combined with 16.7, 8.4, 5.6 or 0 mg/day β-C, respectively. For group E, 5 mg/day VE was administered as a control. After 16 weeks, the cognitive function of the subjects was assessed. The results indicated that, following the treatment intervention, the mean MMSE scores in groups A and B were 23.49±4.40 and 23.44±3.62, respectively, which were significantly higher compared with the score in group E (22.32±4.23; P<0.05; Fig. 1A). For the HDS test, the mean scores in groups A and B following the treatment intervention were 22.46±4.96 and 21.38±3.97, respectively, exhibiting significant elevations compared with the values prior to the treatment (18.68±5.77 for group A and 19.75±5.46 for group B; P<0.05). Furthermore, the HDS scores in groups A and B were also significantly higher than the mean score in group E following treatment (18.87±4.70; P<0.05; Fig. 1B). These results indicate that treatment with VC and VE combined with β-C could markedly improve the cognitive function in the elderly subjects, particularly with higher doses of β-C.

### Effects of VE and VC combined with β-C on the plasma levels of Aβ and E_2_ in the elderly subjects

**Figure 2:**
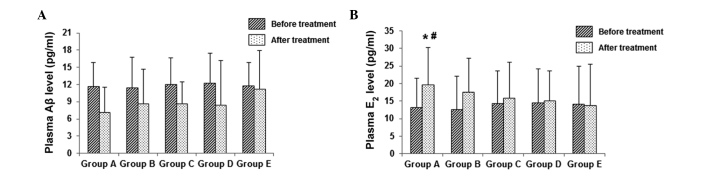
Effect of VE and VC combined with β-C on the plasma levels of (A) Aβ and (B) E_2_ in the elderly subjects prior and subsequent to treatment. Compared with the value prior to the treatment, ^*^P<0.05; compared with the value in group E, ^#^P<0.05. VE, vitamin E; VC, vitamin C; β-C, β-carotene; Aβ, amyloid-β; E_2_, estradiol.

To further investigate the mechanism through which the combination treatments improved the cognitive function in the elderly subjects, the plasma levels of Aβ peptides and E_2_ were measured with RIA. Levels of Aβ peptides have been found to be elevated in patients with AD, which can cause learning and memory impairment in the elderly (13). By contrast, a reduction in E_2_ levels is closely linked to cognitive dysfunction and dementia (14). The results of the present study showed that, following treatment, the plasma Aβ levels in groups A-D were decreased compared with the levels prior to treatment; however, no significant differences were observed (P>0.05; Fig. 2A). For the E_2_ measurement, the plasma E_2_ level in group A following treatment (19.61±10.73 pg/ml) was higher than that prior to the treatment (13.21±8.31 pg/ml; P<0.05), and it was also higher than that in group E subsequent to the treatment (13.72±10.77 pg/ml; P<0.05; Fig. 2B). These results suggest that treatment with VE, VC and β-C could markedly reduce the levels of Aβ and elevate the levels of E_2_ in the plasma in the elderly subjects, which would contribute to the cognitive improvement in these subjects.

## Discussion

Cognitive impairment has been closely associated with free radicals, which can induce cell death, react with cell membranes and cause Aβ deposition (15). Compared with other organs, the brain is more susceptible to free radical damage (16). Under normal physiological conditions, the low level of free radicals produced *in vivo* can be quickly scavenged by the antioxidant defense system, without causing damage to the body. As age increases, the metabolism and enzyme activity in the body undergo changes, and excessive free radicals are generated while the scavenging capacity decreases. An imbalance between free radical generation and clearance could be one of the important factors leading to cognitive dysfunction and neuropathological changes in AD (17).

VE is a fat-soluble antioxidant vitamin, which can efficiently eliminate membrane lipid peroxidation, scavenge oxygen free radicals and maintain the integrity and stability of membranes (18). Furthermore, VE can reduce Aβ toxicity, inhibit the deposition and promote the clearance of the peptide, and alleviate the dysfunction of the brain (19). By contrast, VC, as the major extracellular antioxidant, is the only factor that is capable of preventing the lipid peroxidation induced by water-soluble free radicals (20,21). Furthermore, VC has been found to restore the impaired activity of oxidized VE via the clearance of free radicals. A combination of VE and VC can strengthen the antioxidant defense system (22). Decreased *in vivo* concentrations of VE and VC can result in cellular structural and functional damage, affecting the cognitive function (23). It has been previously shown that the combination of VE and VC improves cognitive function in the elderly population (24).

β-C removes the free radicals in the body, enhances the immune capacity and has been found to prevent cancer, thrombosis and atherosclerosis. An epidemiological survey showed that the age-standardized AD incidence rate in African Americans living in Indianapolis, USA was in excess of two-fold that of native Africans in Ibadan, Nigeria. This difference was attributed to dietary habits. In Ibadan, food was mainly composed of red palm oil and yams, both containing high levels of β-C (25). Consistent with this and the findings from Grodstein *et al* (24) and Martin *et al* (26), the present results indicated that 200 mg/day VE and 300 mg/day VC, combined with high doses of β-C (16.70 or 8.35 mg/day), may improve cognitive function in the elderly.

Aβ is the main component of senile plaques in patients with AD, and is also one of the key factors in the pathology and clinical manifestations of AD (28). Neurotoxic Aβ peptides can destroy the integrity of membranes and increase the intracellular ion permeability. Aβ plaques further activate microglia and trigger inflammatory responses, finally inducing neurodegeneration. Together with oxygen radicals, Aβ can also induce neuronal impairment or even cell death (18). The generation and aggregation of Aβ in the brain has been shown to promote the production of oxygen free radicals and cause damage to neurons (29). Furthermore, the oxidative stress can, in turn, accelerate the production and accumulation of Aβ peptides. In the present study, treatment with VE and VC, combined with β-C, markedly reduced the plasma Aβ levels compared with those prior to treatment, although without significant differences. Further studies are required to clarify the mechanisms through which the investigated combination treatment provided Aβ-decreasing effects in these elderly subjects.

The reduction in E_2_ levels is linked to the cognitive dysfunction in dementia (30). Based on this, E_2_ replacement therapy has been under development to prevent and delay the occurrence of the disease. E_2_ can exert versatile functions in the central nervous system, such as increasing the blood supply to the cerebral cortex, enhancing the uptake and metabolism of glucose in hippocampal neurons, reducing Aβ deposition and reversing neuronal damage (31,32). Furthermore, E_2_ has a high antioxidant activity, which may protect neurons from injury induced by oxidative stress. The present findings showed that treatment with the antioxidants VE, VC and β-C could improve the activity of E_2_, which may have contributed to the cognitive improvement in the elderly population.

In conclusion, the results of the present study showed that combined treatment with VE, VC and β-C could improve the cognitive function of the elderly subjects. Furthermore, the combination treatment decreased the Aβ levels and elevated the E_2_ levels in the plasma from these subjects, which may have contributed to the beneficial effects of the treatment. These findings suggest that VE, VC and β-C administration can result in promising improvements in cognitive function in elderly individuals, and may be suitable for the prevention and treatment of AD.

## Figures and Tables

**Table I:** Background information for the elderly subjects.

						Education	
						
Group	n	Age (years)	Male (n)	Female (n)	Illiterate subjects (n)	Primary school (%)	Secondary school and above (%)
A	56	66.00±5.749	24	32	69.64	26.79	3.57
B	52	67.65±6.013	22	30	69.23	26.92	3.85
C	58	67.88±4.903	26	32	62.07	31.03	6.90
D	55	65.95±5.310	24	31	65.46	27.27	7.27
E	55	67.82±4.402	20	35	69.01	27.27	3.64

Data for age are presented as the mean ± standard deviation.

**Table II:** Baseline analysis of the elderly subjects.

Group	Smoking (n)	Drinking (n)	Hypertension (n)	High blood pressure (n)	Diabetes (n)	Chronic bronchitis (n)	Gastric disease (n)
A	57.14	35.72	12.50	5.36	3.57	10.71	3.57
B	48.08	50.00	13.46	1.92	3.85	7.69	1.92
C	37.93	39.67	10.34	5.17	1.72	12.07	1.72
D	41.82	52.73	9.09	3.64	1.82	7.27	3.64
E	43.64	40.00	10.90	3.64	1.82	9.09	1.82
